# A case of otitis media with effusion due to leiomyoma of the Eustachian tube^☆^

**DOI:** 10.1016/j.bjorl.2017.06.013

**Published:** 2017-07-29

**Authors:** Woo Sub Shim, Young Su Kim, Dong Keun Shin, Hahn Jin Jung

**Affiliations:** Chungbuk National University Hospital, Chungbuk National University College of Medicine, Department of Otorhinolaryngology-Head and Neck Surgery, Cheongju, Republic of Korea

## Introduction

Otitis media with effusion (OME) has several potential causes and the Eustachian tube dysfunction is one of the leading causes.^1^ There are essentially two types of the Eustachian tube obstruction resulting in OME: mechanical and functional. Mechanical obstruction may be either intrinsic or extrinsic. Intrinsic mechanical obstruction is usually caused by inflammation of the mucous membrane lining of the Eustachian tube or an allergic diathesis causing edema of the tubal mucosa. Extrinsic mechanical obstruction is caused by obstructing masses such as adenoid tissue or nasopharyngeal neoplasm.^2^

Leiomyoma is a benign tumor of smooth muscle origin and the commonest site of leiomyoma is uterus followed by skin and gastrointestinal tract. It can arise from any tissue containing non striated muscles, but leiomyoma of head and neck region account for less than one percent of all leiomyomas because those regions lack smooth muscle.^3^ The leiomyoma originating from the Eustachian tube and causing otitis media with effusion due to intrinsic obstruction of Eustachian tube has never been reported.

Here we describe an interesting case of OME due to leiomyoma arising from inside of the Eustachian tube which was excised completely by endoscopic endonasal approach with review of the literature.

## Case report

A 35 year-old man presented with a 6 year history of recurrent left OME. He had complaints about left aural fullness and hearing disturbance. He had those symptoms every time when he had acute pharyngolaryngitis and the symptoms subsided with cold medications. But this time, left aural fullness went on for 3 months despite the treatment. He had no history of ventilation tube insertion, and had tonsillectomy without adenoidectomy 10 years ago. He did not have otalgia, otorrhea, hyperacusis, autophonia, rhinorrhea, and postnasal drip.

**Figure 1:**
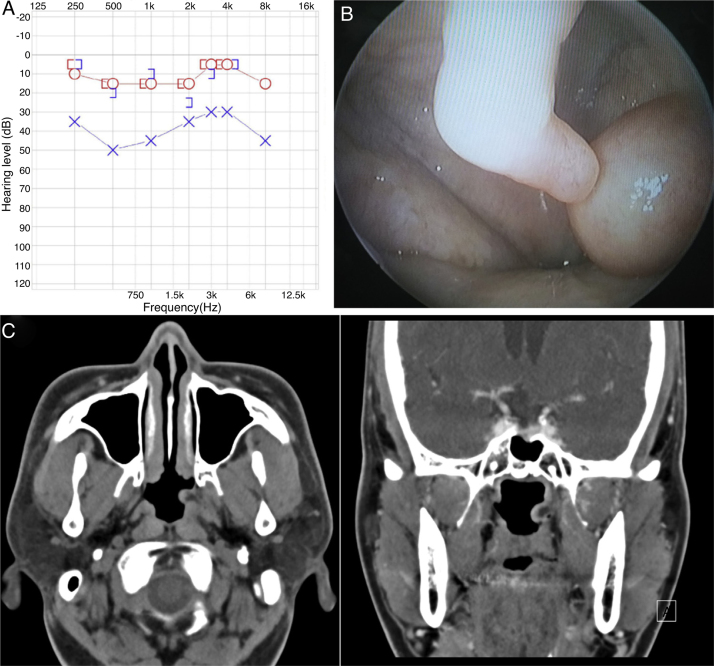
Preoperative findings. (A) Pure tone audiometry had 24 dB conductive hearing loss in the left ear. (B) Nasopharyngoscopic examination showed an about 10 × 5 smooth surfaced, round soft mass in the left side of nasopharynx, obstructing the opening of the left Eustachian tube totally. (C) Contrast-enhanced PNS CT shows a smooth-margined, mild enhancing, exophytic lesion in the opening of the left Eustachian tube. (left) Axial section, (right) coronal section.

On physical examination, otoscopy revealed effusion in the middle ear and turbid tympanic membrane. Pure tone audiometry had 24 dB conductive hearing loss in the left ear (Fig. 1A) and tympanometry found ‘B’-type curve in the left ear. Nasopharyngoscopic exam displayed an about 10 × 5 mm smooth-surfaced, round soft mass in the left side of nasopharynx, obstructing the opening of the left Eustachian tube totally (Fig. 1B). Contrast-enhanced computed tomography was done, which showed a smooth-margined, exophytic growing lesion with mild enhancement involving the left Eustachian tube, measuring 10 × 6 mm (Fig. 1C).

**Figure 2:**
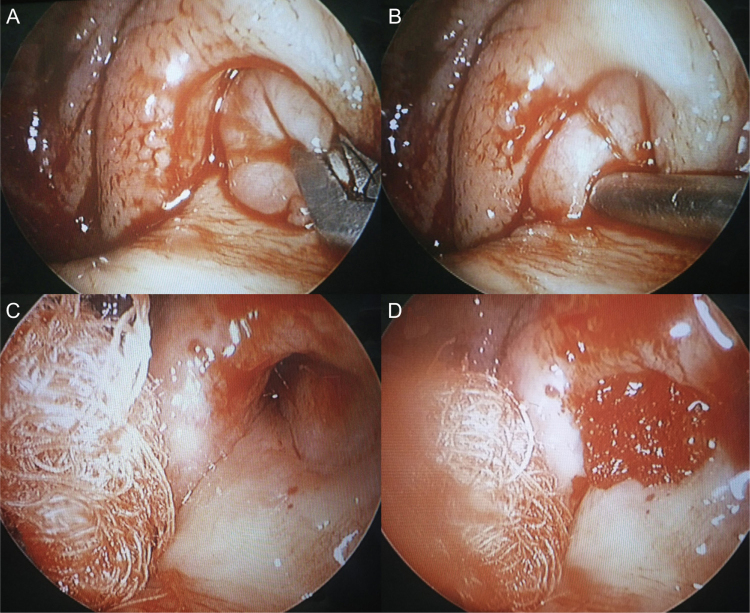
Intraoperative findings. (A and B) The mass rised from the pharyngeal orifice of the Eustachian tube and extended to the nasopharynx. (C and D) After complete removal via endoscopic approach, Gelfoam® was soaked in dexamethasone and gentamycin and inserted in orifice where there was a stalk of the mass.

Round mass was removed completely and uneventfully via endoscopic endonasal approach. The mass rised from the pharyngeal orifice of the Eustachian tube and extended to the nasopharynx (Fig. 2A and B). The intraoperative frozen-section showed to be benign lesion without malignancy. Stalk, about 5 mm inside the Eustachian tube was removed completely with cutting forceps. Bleeding was minimal. Normal mucosa was not damaged and the Eustachian tube orifice was carefully preserved to prevent synechia or stenosis postoperatively. Gelfoam® was soaked in dexamethasone and gentamicin and inserted in orifice where there was a stalk of the mass (Fig. 2C and D). The myringotomy was done to the left tympanic membrane and mucoid discharge was drained.

**Figure 3:**
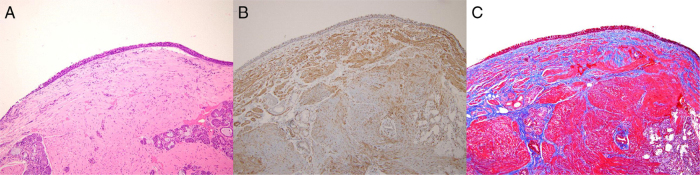
Pathologic findings (100×). (A) Specimen shows a well circumscribed lesion having spindle shaped cells arranged in whorls and fascicles with eosinophilic cytoplasm in hematoxylin and eosin stain. (B) Smooth muscle actin staining revealed smooth muscle component (black). (C) Masson trichrome staining exhibited smooth muscle cells (red) with intervening collagen fibers (blue).

**Figure 4:**
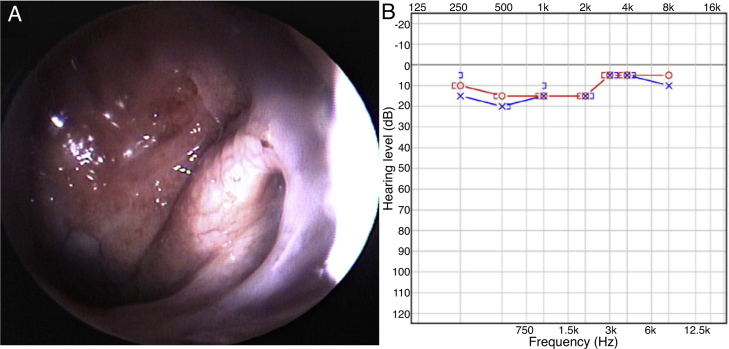
Postoperative findings. (A) Nasopharyngoscopy showed that the mass was removed and the Eustachian tube orifice remained wide open. (B) Pure tone audiogram showed that the hearing of left ear was normalized and air-bone gap was disappeared.

About two week after operation, his symptoms disappeared. The histopathological examination of the excised mass showed features suggestive of leiomyoma, composed of well-differentiated smooth muscle cells confirmed by smooth muscle actin and Masson's trichrome stain. Mitoses were not found, and nuclear atypia was absent (Fig. 3). After the surgery, Eustachian tube orifice remained wide open and pure tone audiometry was normalized without air-bone gap (Fig. 4). On 12 months follow up, patient is fine without any recurrence of mass and symptoms of OME.

## Discussion

We demonstrated a case of leiomyoma at the Eustachian tube orifice, resulting in recurrent OME. To the best of our knowledge, this is the first case of a leiomyoma originating from the Eustachian tube, and removed successfully by endoscopic endonasal approach.

Nasopharyngeal tumors present as unilateral nasal obstruction, aural fullness, hearing loss and epistaxis. Diagnosis is usually achieved by rhinopharyngoscopy and radiological investigations; however, histopathological examination confirms it. Adenoid is the most common soft-tissue tumor in the nasopharynx. Antrochoanal polyp, inverted papilloma and pleomorphic adenoma are the few other benign tumors of the nasopharynx but leiomyoma is an extremely rare tumor in the nasopharynx.^4^

Leiomyoma is a benign tumor of smooth muscle origin that can occur at anywhere in the body containing non-striated muscles. Leiomyoma is defined by the World Health Organization as a “circumscribed benign, often cutaneous tumor composed of intersecting bundles of mature smooth muscle cells.” The frequent sites of leiomyoma are female genital tract (95%) followed by skin (3%), and gastrointestinal (1.5%). Leiomyoma of head and neck region account for less than one percent of all leiomyomas,^5^ and Farman et al. reviewed 7748 cases of smooth muscle tumors involving whole body and reported that only 5 cases involving the oral cavity was found.^6^ Davis et al. mentioned a total of 139 cases of leiomyoma of the oral cavity and pharynx, and the most common sites of leiomyoma of the oral cavity and pharynx were the lips (27.46%), tongue (18.30%), and hard and soft palate.^7^ And Fu and Perzin reported that only two leiomyomas were found in a series review of 256 non-epithelial neoplasms of the sinonasal tract and nasopharynx.^8^ This is the first case report of leiomyoma found in nasopharynx originating from the Eustachian tube. Leiomyoma presents as a nodular swelling of varying color depending upon the vascularity and slowly increases in size. These tumors generally present as painless masses and symptoms are caused by the mass effect and the diagnosis is established only by histopathological examination with special stains for smooth muscle component.^6^ In this case, due to mass effect, recurrent OME was developed. Clinically, connective tissue tumors e.g. fibromas, lipomas may present similar findings to leiomyoma; hence a differential diagnosis must be established. The differential diagnosis, moreover, must also include the malignant form of leiomyoma, i.e. leiomyosarcoma. Usually the surgical excision is the treatment of choice. Recurrences are not common after complete excision.^9^ In this case, the tumor has been successfully removed by endoscopic endonasal approach, and no recurrence has been found up till now.

It is not uncommon to have OME due to the Eustachian tube dysfunction. The causes are extrinsic obstruction (adenoid hypertrophy, nasopharyngeal tumor, etc.), inflammation of the mucous membrane lining of the Eustachian tube (due to upper respiratory infection, pharyngolaryngeal reflux, allergy, smoking, etc.), intrinsic mechanical obstruction (middle ear cholesteatoma, polypoid mucosa of middle ear, etc.), and functional obstruction (craniofacial anomaly, dysfunction of tensor veli palatine muscle, etc.). Therefore, an adult with unilateral OME should undergo thorough examination including nasopharyngoscopy to rule out the presence of an unusual underlying pathologic condition. In this case, nasopharynx examination was not done despite the 6 years recurrent OME. With adequate local anesthesia and shrinkage, nasopharynx exam can be easily performed in the outpatient setting with nasopharyngoscopy. Our case points out the necessity of a nasopharyngoscopic examination for patients who present with recurrent OME.

The surgical approach involving the Eustachian tube is a difficult procedure because of the inadequate exposure. Transmandibular, transmaxillary, transpalatal and transpterygoid approaches have been used for larger tumors. Nowadays with the advent of transnasal endoscopic surgery, more and more tumors located in the nasopharynx and lower part of the Eustachian tube have been treated in this way. For our case, an endoscopic endonasal approach was the best choice considering the tumor was contained within the orifice of the Eustachian tube and nasopharynx. The use of endoscopic techniques provided an excellent visualization and identification of attachment site inside the Eustachian tube, thereby could avoid mucosal trauma and its sequelae. The alternative approach could have been transpalatal approach but it carries a risk of fistula formation along with post-operative pain. Our case did not have any postoperative pain and was discharged on the operative day.

## Conclusion

This is the first case of Eustachian tube leiomyoma to be reported in the literature that emphasizes the role of nasal endoscopy and rarity of this tumor. The Eustachian tube leiomyoma could be removed successfully via endoscopic endonasal approach with less post-operative morbidity and early recovery.

## Conflicts of interest

The authors declare no conflicts of interest.
